# Effects of testosterone treatment on clitoral haemodynamics in women with sexual dysfunction

**DOI:** 10.1007/s40618-021-01598-1

**Published:** 2021-06-12

**Authors:** S. Cipriani, E. Maseroli, V. Di Stasi, I. Scavello, T. Todisco, G. Rastrelli, M. Fambrini, F. Sorbi, F. Petraglia, E. A. Jannini, M. Maggi, L. Vignozzi

**Affiliations:** 1 Andrology, Women’s Endocrinology and Gender Incongruence Unit, Department of Experimental, Clinical and Biomedical Sciences “Mario Serio”, University of Florence, Viale Gaetano Pieraccini 6, 50139 Florence, Italy; 2 Department of Experimental, Clinical and Biomedical Sciences “Mario Serio”, Gynecology Unit, University of Florence, Florence, Italy; 3 Endocrinology and Medical Sexology (ENDOSEX), Department of Systems Medicine, University of Rome Tor Vergata, Rome, Italy; 4 Endocrinology Unit, Department of Experimental, Clinical and Biomedical Sciences “Mario Serio”, University of Florence, Florence, Italy; 5 I.N.B.B. (Istituto Nazionale Biostrutture E Biosistemi), Rome, Italy

**Keywords:** Female sexual dysfunction, Clitoris, Ultrasound, Testosterone treatment in women, Treatment for female sexual dysfunction, Genital blood flow

## Abstract

**Purpose:**

To explore the effects of 6-month systemic testosterone (T) administration on clitoral color Doppler ultrasound (CDU) parameters in women with female sexual dysfunction (FSD).

**Methods:**

81 women with FSD were retrospectively recruited. Data on CDU parameters at baseline and after 6 months with four different treatments were available and thus further longitudinally analyzed: local non-hormonal moisturizers (NH group), *n* = 37; transdermal 2% T gel 300 mcg/day (T group), *n* = 23; local estrogens (E group), *n* = 12; combined therapy (T + E group), *n* = 9. Patients underwent physical, laboratory, and genital CDU examinations at both visits and completed different validated questionnaires, including the Female Sexual Function Index (FSFI).

**Results:**

At 6-month visit, T therapy significantly increased clitoral artery peak systolic velocity (PSV) when compared to both NH (*p* < 0.0001) and E (*p* < 0.0001) groups. A similar increase was found in the T + E group (*p* = 0.039 vs. E). In addition, T treatment was associated with significantly higher FSFI desire, pain, arousal, lubrication, orgasm, and total scores at 6-month visit vs. baseline. Similar findings were observed in the T + E group. No significant differences in the variations of total and high-density lipoprotein-cholesterol, triglycerides, fasting glycemia, insulin and glycated hemoglobin levels were found among the four groups. No adverse events were observed.

**Conclusion:**

In women complaining for FSD, systemic T administration, either alone or combined with local estrogens, was associated with a positive effect on clitoral blood flow and a clinical improvement in sexual function, showing a good safety profile.

**Trial registration number:**

NCT04336891; date of registration: April 7, 2020.

## Introduction

Female sexual dysfunction (FSD) is a multifactorial condition in which organic, relational, and psychosocial factors are deeply intertwined. Among organic factors, hormonal unbalance has a major impact on FSD development. In particular, a subtle and progressive age-dependent decline of androgen levels has been implicated in the pathogenesis of both Hypoactive Sexual Desire Disorder (HSDD) [1] and Genitourinary Syndrome of Menopause (GSM) [2], whose prevalence increases with age [3, 4]. However, the diagnosis of androgen deficiency in women is controversial. In 2014, the Endocrine Society Clinical Practice Guideline recommended against making a clinical diagnosis of androgen deficiency syndrome in healthy women, since there is a lack of well-defined criteria [5], as opposed to men [6]. In fact, a biochemical definition of female androgen deficiency is not completely reliable, because of the lack of standardized, accurate assays for androgens at the low levels characteristic of women and, as well, the lack of valid reference ranges [5]. Nevertheless, when considering an evidence-based-medicine approach to this topic, several findings count in favour of the existence of a clinically manifest hypoandrogenism in women. Postmenopausal women reporting low sexual desire have represented the main study population so far taken into account to demonstrate that female sexual function is a target of androgen action [5]. According to the definition of HSDD developed by the International Society for the Study of Women Sexual Health (ISSWSH) nomenclature committee, this condition can manifest as any of the following for a minimum of 6 months: lack of motivation for sexual activity (decreased/absent spontaneous or reactive to erotic stimulation desire); loss of desire to initiate or participate in sexual activity not as a consequence of sexual pain disorders; combination with clinically significant personal distress [1]. Strong clinical evidence supports the use of testosterone (T) treatment for HSDD in postmenopause [3], whilst only some studies describe its clinical efficacy also in peri- and premenopausal patients [7]. Specifically, it has been reported that T treatment significantly influences multiple domains of sexual functioning, by improving desire, arousal, lubrication, pain, orgasm, and satisfaction [8–11].

In hypogonadal men, the use of T replacement therapy (TRT) is currently well established and it is strongly recommended to induce/maintain secondary sex characteristics and to correct symptoms of T deficiency [12]. The molecular pathways underlying the effects of T on male sexual function have been largely elucidated; in particular, the erectile response to T is mediated at both central (through an effect on sexual desire) and peripheral (through the effect of vasorelaxant mechanisms on penile corpora cavernosa) level [13–15]. In hypogonadal men with erectile dysfunction, TRT was able to improve penile vasodilation as assessed using color Doppler ultrasound (CDU) [16, 17]. In contrast, despite the clinical evidence and the current consensus on the central effect of T on female sexual desire [3, 18], the mechanisms by which androgens may directly act in relevant female brain areas have not been elucidated [19]. Recently, a study from our group demonstrated that the androgen receptor (AR) super-agonist dihydrotestosterone (DHT), which is not aromatizable to estrogen, was able to stimulate sexual behaviors in ovariectomized female rats [19], therefore suggesting that conversion into estrogens is not required for the facilitatory effect of androgens on sexual desire [19]. In vivo systemic T treatment in ovariectomized rats also improves the relaxation of clitoral vascular smooth muscle cells (SMCs) through the nitric oxide (NO)—cyclic guanosine monophosphate (cGMP) pathway, thus sustaining the major relaxant mechanisms involved in genital sexual arousal [20]. These findings strongly indicate that T could exert a positive peripheral effect on sexual response also in females, as previously observed in the male gender. However, clinical studies aimed at evaluating whether T treatment could improve clitoral blood flow are still lacking.

For these reasons, we conducted a pilot explorative research on women seeking medical care for HSDD and treated with systemic T. The primary aim of the study was to evaluate whether T treatment is associated with a change in peak systolic velocity (PSV) of clitoral artery as assessed by CDU and whether this change is different from that observed in other treatment regimens. Psychosexual, biochemical, and metabolic parameters were also evaluated to assess the clinical efficacy and cardio-metabolic safety of T treatment.

## Methods

The present study is an observational, retrospective analysis of pre- and postmenopausal women who attended our outpatient clinic at Andrology, Women’s Endocrinology and Gender Incongruence Unit, University of Florence (Florence, Italy) for sexual concerns, from March 2019 to March 2020. The protocol is in accordance with the Declaration of Helsinki and was approved by the local Ethics Committee (protocol TESTOFSD, 226/5/2019, 14457/OSS, Careggi Hospital, Florence, Italy) and registered in the U.S. National Library of Medicine database (NCT04336891). Informed consent was obtained before the initiation of any clinical procedure. The primary outcome of the study was to evaluate the difference in the change of clitoral artery PSV among the four treatment groups (see below).

### Subjects

For the purpose of the study, only women fulfilling the following criteria were included in the analysis: age ≥ 18 years, being heterosexual, sexually active in the last 6 months, diagnosed with FSD, without a history of drug or alcohol abuse and without a diagnosis of uncontrolled or unstable mental or organic diseases. Therefore, a consecutive series of 81 women who had received as per clinical practice one of the following treatments for at least 6 months was considered:

- women with dyspareunia due to mild to moderate vulvovaginal atrophy (VVA), with contraindications to hormonal therapy or who wished to avoid it, treated with non-hormonal moisturizers applied regularly every 2–3 days and lubricants as needed (non-H therapy group; *n* = 37);
- women with HSDD treated with *off-label* transdermal 2% T gel applied once daily to the thighs or lower abdominal/pubic area (300 mcg T per day) (transdermal T group; *n* = 23);
- women with dyspareunia due to moderate to severe VVA, treated with local estrogens (estradiol or promestriene ovules) taken daily for 2 weeks and afterwards twice a week (E group; *n* = 12);
- women with HSDD reporting also significant dyspareunia due to moderate to severe VVA, treated with combined therapy (transdermal T and local estrogens) (combined, T + E group; *n* = 9). In all other cases of overlapping FSD diagnoses, a common finding in clinical practice, the patient was categorized according to the dysfunction which was reported as the more distressing, or which was identified by the physician as the first to have been developed.

Among the subjects included in the analysis, there were no patients who dropped out or switched therapy over the 6-months follow-up. HSDD was diagnosed according to the ISSWSH consensus nomenclature [21]. In women with dyspareunia due to VVA, local dermatologic diseases, infections, trauma associated with genital surgery or radiotherapy, lesions and tumors were evaluated and excluded during gynecological examination.

### General baseline assessment

At baseline, for all (*n* = 81) women enrolled in the study we collected demographic data, sexual and relational history, obstetric/gynecological history, as well as information on education, smoking and drinking habits, physical exercise, medications and associated medical and psychiatric conditions. Additionally, body weight, height, body mass index (BMI), waist circumference, systolic and diastolic blood pressure were registered to evaluate the metabolic status and the presence of cardiovascular (CV) risk factors. Finally, all patients underwent a CDU as well as biochemical and psychometric evaluation.

### Color Doppler ultrasound assessment

CDU of clitoral and labial arteries was carried out by an experienced operator blinded to the clinical data using the MyLabClass-C sonography system (Esaote SpA, Genova, Italy) with a linear (LA523, 6–13 MHz) transducer, as previously described [22]. In premenopausal women, CDU was performed during the early follicular phase of the menstrual cycle (days 3–5) to ensure standardized conditions. All women were scanned in a quiet room with stable conditions of heating and lighting to decrease the impact of external factors on blood flow. CDU was performed according to previously reported procedures [22–24]. Briefly, we asked participants to abstain from sexual activity, including masturbation, for at least 12 h before examination, and to void their bladder immediately before to guarantee standardized conditions. Patients were scanned in the lithotomy position (the patient lies on her back with her legs well separated, thighs flexed on the abdomen, and calves on thighs), with a sufficient quantity of sonographic jelly to avoid interference from air and without applying any significant pressure on the genital tissues, to minimize possible artifacts [25, 26]. The clitoral cavernous arteries, appearing at the center of each clitoral body, are easily localized by placing the transducer transversally, on the top of the vulva, while the posterior labial artery (a branch of the internal pudendal artery) is visualized by placing the transducer in a longitudinal plane, postero-laterally to the labia majora (at about 2 cm from the clitoral hood) [23, 27]. When adequate Doppler signals are detected, pulse-wave Doppler mode is activated, and blood flow velocity waveforms are recorded. For both clitoral and labial arteries, at least three sequential waveforms are obtained for each side, to define mean values of the considered hemodynamic parameters. For clitoral and labial arteries the following hemodynamic parameters were automatically computed: PSV, acceleration (ACC) and pulsatility index (PI), which represents the difference between the peak systolic and the end-diastolic flows divided by the mean maximum flow velocity [28]. The PI has been reported to reflect resistance to blood flow [29, 30].

### Biochemical parameters

As part of their clinical visit for evaluation of sexual symptoms, women were asked to have blood samples drawn in the morning, after an overnight fast, during the early follicular phase (if premenopausal), to measure the following parameters: E_2_ (17β-estradiol) (Luminescent Oxygen Channeling Immunoassay LOCI, Dimension Vista Siemens, Berlin, Germany); DHEAS (dehydroepiandrosterone) and SHBG (sex hormone binding globulin) (ElectroChemiLuminescence ImmunoAssay ECLIA, COBAS 600 Roche Diagnostics, Basel, Switzerland); A4 (Δ4-androstenedione, Radio ImmunoAssay RIA, Access 2 Beckman Coulter, USA); total testosterone (TT) (chemiluminescence immunoassay; Advia Centaur, Siemens, Berlin, Germany); fasting glucose (glucose hexokinase method; Dimension Vista 1500, Medical Solutions Siemens Healthcare, Malvern, PA, USA); total cholesterol, HDL (High-Density Lipoprotein-cholesterol) and triglycerides (automated enzymatic colorimetric method; Dimension Vista 1500, Medical Solutions Siemens Healthcare); fasting insulin (electrochemiluminescence immunoassay; Roche Diagnostics, Mannheim, Germany) and HbA1c (glycated hemoglobin, high-performance liquid chromatography; Variant II, Bio-Rad Laboratories, Hercules, CA, USA). LDL (low-density lipoprotein-cholesterol) was calculated indirectly by the Friedewald equation, LDL = total cholesterol—(HDL + triglycerides/5), where all parameters are expressed in milligrams per deciliter. Calculated free T (cFT) was estimated according to the formula of Vermeulen et al. (available at http://www.issam.ch/freetesto.htm). The homeostatic model assessment (HOMA) used to quantify insulin resistance and β-cell function was calculated based on the following approximation formula: HOMA for insulin resistance = (glucose × insulin)/22.5, where glucose and insulin are expressed as millimoles per liter. The actual calculated HOMA2 compartmental model is published and is available as the interactive Homeostatic Model Assessment 2 (iHOMA2; available at: http://www.phc.ox.ac.uk/research/technology-outputs/ihoma2).

### Metabolic syndrome assessment

Metabolic syndrome (MetS) was diagnosed according to the National Cholesterol Education Program Adult Treatment Panel III [31], considering the presence of at least three of the following five factors at first visit: central obesity (waist circumference > 88 cm), increased triglycerides level (≥ 150 mg/dL or treatment for hypertriglyceridemia), increased blood pressure (systolic blood pressure ≥ 130 mm Hg and/or diastolic blood pressure ≥ 85 mm Hg or antihypertensive treatment), increased fasting glucose level (≥ 110 mg/dL or anti-diabetic treatment), and decreased HDL level (< 50 mg/dL or treatment for dyslipidemia).

### Psychosexual assessment

Sexual and relational data were derived by a standardized interview described in detail by Maseroli et al. [32]. The frequency of sexual events was assessed using a standard question (‘During the last 3 months, how many times on average did you engage in sexual activity in a month?’), scoring 0 = no events, 1 = 1–2 events, 2 = 3–7 events and 3 =  ≥ 7 events monthly.

All patients filled out the Female Sexual Function Index (FSFI) [33] in its validated Italian version [34]. The FSFI evaluates all phases of the female sexual response (desire, arousal, and orgasm), sexual satisfaction, and dyspareunia. Single scores for each question range from a minimum of 0 or 1 to a maximum of 5. Domain scores result from adding individual question scores and multiplying them for a specific factor. The total score is obtained by adding the six domain scores. Women with a total score lower than 26.55 are classified as being at risk for FSD.

In addition, data deriving from the Middlesex Hospital Questionnaire (MHQ), a brief self-administered questionnaire for the screening of mental disorder symptoms in a non-psychiatric setting [35], were recorded. The MHQ provides a total index of psychopathology (MHQ total score) and scores for free-floating anxiety (MHQ-A), phobic anxiety (MHQ-F), obsessive–compulsive traits and symptoms (MHQ-O), somatization (MHQ-S), depressive symptoms (MHQ-D), and histrionic or hysterical symptoms (MHQ-I).

Finally, we collected data from the Body Uneasiness Test (BUT), which is designed to explore body uneasiness and dissatisfaction [36], which was completed at both visits. This self-reported questionnaire includes questions regarding 34 body experiences (BUT-A) and dissatisfaction with 37 body parts (BUT-B). The total average score of the BUT-A indicates the degree of severity related to body image and it is expressed by the Global Severity Index (GSI). A woman is considered at risk of discomfort with her body if the GSI score is higher than 1.2. BUT-A subscales define dissatisfaction regarding the body and its weight (WP), avoiding behavior (AV), compulsive self-monitoring (CSM), experience of depersonalization (DEP), including separation and foreignness regarding the body and body image concerns (BIC). Answers are scored on a six-point Likert-type scale (from “never” to “always”), with higher scores denoting greater body uneasiness. BUT-B scores, related to dissatisfaction with different body parts, come together into two global measurements: positive symptoms total (PST) and positive symptoms distress index (PSDI).

### 6-month visit analysis

When analyzing the patients’ medical records, following data registered both at baseline and at 6-month visit were available and were collected:

- PSV, ACC and PI of clitoral arteries, to investigate changes in genital blood flow;
- clitoral area (only in women treated with T), to investigate T biological effects and safety;
- FSFI total and subdomains scores, to investigate clinical efficacy;
- TT and SHBG levels (only in women treated with T), to evaluate compliance and check for overdosing;
- total cholesterol, HDL, triglycerides, HbA1c, fasting glucose, and insulin levels, to investigate cardio-metabolic safety.

Adverse events were monitored according to routine practice, through physical and gynecological examination, interviews, transvaginal ultrasound and biochemical analysis whenever necessary.

### Statistical analysis

Data were expressed as mean ± standard deviation (SD) when normally distributed and as median (quartile) for parameters with non-normal distribution, unless otherwise specified.

Mann–Whitney *U* test and Student’s *t* test were used for comparisons of not normally and normally distributed continuous variables between two independent groups, respectively. Kruskal–Wallis test and one-way ANOVA were performed to analyze the differences between the medians of not normally and normally distributed variables between more than two independent groups, respectively. These statistical analyses were performed using SPSS 24.0 for Windows (SPSS Inc, Chicago, IL, USA).

For the analyses on CDU, sexual and serum parameters, the change over time in the treatment groups was assessed by the multilevel mixed-effects linear regression using the treatment group, the time-points and their interaction as independent variables, the measure of the variable at 6-month visit as the dependent variable and the patients’ ID as random effect. The analyses were adjusted for the first visit value of the dependent variable along with age and years since menopause. For each parameter, the estimated mean change (EMC) and the 95% confidence interval (CI) with the *p* value between groups or within the same group were reported. These statistical analyses were conducted using Stata MP 13·1 for Windows (StataCorp, College Station, TX, USA).

## Results

### Baseline analysis

The baseline characteristics of patients, divided into four groups according to the different treatments (non-hormonal therapy, *n* = 37; transdermal T, *n* = 23; E, *n* = 12; combined T + E, *n* = 9), are reported in Table 1. We did not observe significant differences in the majority of the investigated parameters among the four groups, with the exception of age and HDL value. Indeed, women receiving transdermal T were older and had higher HDL levels than those receiving non-hormonal therapy (51.74 ± 8.72 vs. 41.84 ± 14.20 years, *t* = 3.011, *p* = 0.004, for age; 69.52 ± 18.08 vs. 54.28 ± 14.29 mg/dL, *t* = 3.207, *p* = 0.003, for HDL) (Table 1). Moreover, we observed a statistically significant difference in the percentage of post-menopausal women among the four groups (*p* = 0.016); therefore, further analyses were adjusted for years since menopause.

**Table 1:** Baseline demographic, metabolic, hormonal, and CDU characteristics of the sample

	*n* = 37	*n* = 23	*n* = 12	*n* = 9	*p*
	Non-H therapy	Transdermal T	E	Combined (T + E)	
Demographic and metabolic parameters					
Age (years)	41.84 ± 14.20	51.74 ± 8.72	45.58 ± 15.52	50.00 ± 16.50	**0.004**
Menopause, % (*n*)	27.03 (10)	65.22 (15)	58.33 (7)	55.56 (5)	**0.016**
BMI (kg/m^2^)	27.48 ± 8.29	26.89 ± 5.01	23.66 ± 3.59	23.64 ± 3.28	0.188
Waist (cm)	100.20 ± 20.61	99.18 ± 14.58	92.36 ± 11.65	88.14 ± 12.62	0.268
Total cholesterol (mg/dL)	197.45 ± 43.36	213.76 ± 28.37	226.91 ± 28.67	197.38 ± 30.17	0.089
HDL-cholesterol (mg/dL)	54.28 ± 14.29	69.52 ± 18.08	73.64 ± 27.45	62.57 ± 13.79	**0.003**
Triglycerides (mg/dL)	95.00 [73.50–138.25]	99.50 [67.50–129.00]	88.00 [60.00–151.00]	70.00 [42.00–79.00]	0.176
LDL-cholesterol (mg/dL)	122.19 ± 38.53	123.54 ± 21.29	133.27 ± 38.02	115.46 ± 30.46	0.707
Fasting glycemia (g/L)	0.95 ± 0.18	0.89 ± 0.10	0.88 ± 0.11	0.98 ± 0.39	0.412
Fasting insulin (mU/L)	11.05 [4.32–23.17]	5.80 [4.20–11.20]	5.90 [4.90–8.70]	6.00 [4.15–11.40]	0.577
HOMA-IR (U)	3.82 ± 3.63	2.13 ± 1.76	1.35 ± 0.46	1.34 ± 0.63	0.117
HbA1c (mmol/mol)	40.37 ± 8.43	35.44 ± 4.67	38.38 ± 5.09	36.20 ± 12.40	0.225
Hormonal parameters					
SHBG (nmol/L)	59.20 [42.65–86.95]	57.20 [39.20–120.10]	74.65 [57.95–167.30]	78.00 [53.95–128.75]	0.178
Total testosterone (nmol/L)	0.80 [0.37–1.55]	0.80 [0.45–1.29]	0.50 [0.40–1.30]	0.69 [0.42–1.27]	0.444
cFT (pmol/L)	9.87 [6.40–20.45]	7.91 [4.98–15.60]	4.48 [2.92–15.97]	5.52 [3.21–12.89]	0.437
17β-estradiol (pmol/L)	121.00 [42.50–228.25]	68.00 [23.50–246.32]	143.04 [60.00–166.75]	181.77 [33.38–267.06]	0.232
Clitoral CDU parameters					
Clitoral PI	1.60 [1.10–2.14]	1.58 [1.26–2.47]	1.66 [1.05–2.17]	1.29 [0.83–1.76]	0.775
Clitoral artery peak systolic velocity (cm/s)	9.20 [7.00–11.80]	7.70 [5.20–8.80]	9.20 [6.32–10.38]	6.80 [5.35–9.05]	0.116
Clitoral artery acceleration (m/s^2^)	1.21 [0.61–2.23]	0.98 [0.61–1.71]	0.98 [0.44–2.75]	0.52 [0.22–1.11]	0.317

Data are expressed as mean ± standard deviation when normally distributed and as median [quartile] when not normally distributed. Bold indicates statistically significant difference between the four groups (*p* < 0.05)

*BMI* body mass index, *CDU* color Doppler ultrasound, *E* estrogens, *HbA1c* glycated hemoglobin, *HDL* high-density lipoprotein, *HOMA-IR* homeostasis model assessment-insulin resistance, *LDL* low-density lipoprotein, *Non-H* non-hormonal, *SHBG* sex hormone binding globulin, *cFT* calculated free testosterone, *PI* pulsatility index, *T* testosterone

**Fig. 1:**
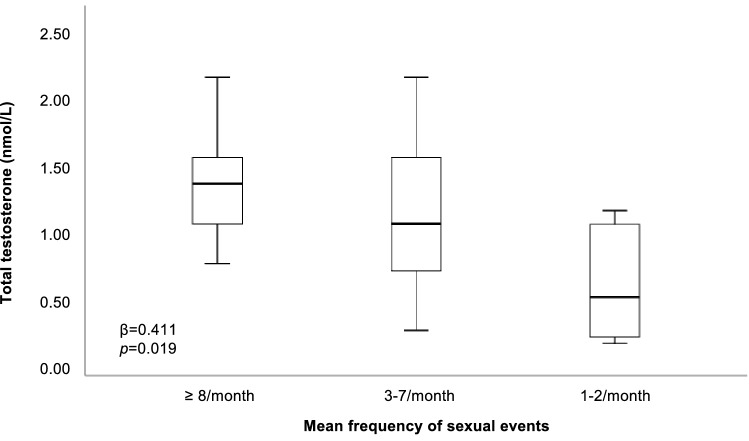
Total testosterone levels according to the mean frequency of sexual events in sexually active women treated for FSD. Data are expressed as box plot, with median score and interquartile range; whiskers represent minimum and maximum values. Data were adjusted for age and years since menopause

We did not observe any association between TT and FSFI scores (not shown). However, TT levels significantly and positively correlated with the mean frequency of sexual events, even after adjusting for age and years since menopause (*β* = 0.411, *p* = 0.019) (Fig. 1). No significant association was observed between TT, DHEAS, A4, or cFT with genital CDU parameters (not shown).

### Effects of transdermal testosterone treatment on clitoral CDU parameters

**Fig. 2:**
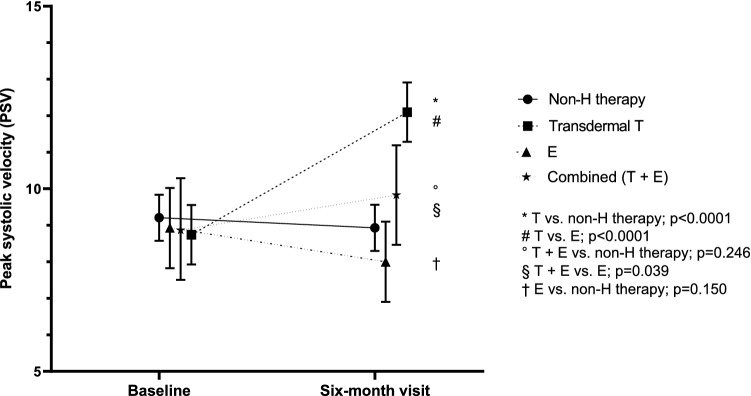
Effect of different treatments for female sexual dysfunction on clitoral artery PSV. Data are derived from multilevel mixed-effects linear regression. Results are reported as estimated PSV value and 95% confidence interval at baseline and at 6-month visit in the four treatment groups. Meaning of asterisks is reported in figure as inset. *PSV* peak systolic velocity, *Non-H* non-hormonal, *T* testosterone, *E* estrogens

The effects of 6-month treatment on PSV, as a change between 6-month visit and baseline, are reported in Fig. 2. More in detail, T treatment was able to significantly increase clitoral PSV when compared to both non-hormonal therapy (EMC 3.17 cm/s [2.12; 4.22], *p* < 0.0001) and local E (EMC 4.10 cm/s [2.73; 5.47], *p* < 0.0001) (Fig. 2). A significant increase in clitoral PSV vs. E was found also in patients on combination therapy (T + E) (EMC 1.83 cm/s [0.09; 3.57], *p* = 0.039) (Fig. 2). Conversely, patients treated only with E showed a reduction in PSV, although the variation was not significant as compared to non-hormonal therapy (EMC − 0.93 cm/s [-2.20; 0.34], *p* = 0.150) (Fig. 2). On the contrary, no significant differences were found in the percentage of change of clitoral ACC and PI among the four groups (not shown).

### Effects of testosterone treatment on sexual parameters

Interestingly, at 6-month visit, patients treated with transdermal T showed significantly higher FSFI desire, pain, arousal, lubrication, orgasm and total scores as compared to baseline (Table 2). A change was also observed for FSFI satisfaction score, approaching statistical significance (Table 2). Similarly, patients receiving combined therapy (T + E) showed significantly higher FSFI desire, arousal, lubrication and total scores as compared to baseline (Table 3). Finally, T + E treatment group showed a numerical change in FSFI overall pain and satisfaction scores, approaching statistical significance, while the change in the orgasm score was not significant (Table 3).

**Table 2:** Change over 6-months follow-up in the scores of FSFI domains in the T treatment group

FSFI domain	Estimated mean change [95% CI]	*p*
Desire	1.24 [0.69–1.79]	** < 0.001**
Pain	0.90 [0.01–1.79]	**0.047**
Arousal	1.45 [0.81–2.08]	** < 0.001**
Lubrication	1.32 [0.48–2.17]	**0.002**
Orgasm	1.35 [0.59–2.11]	** < 0.001**
Satisfaction	1.78 [− 0.21–3.76]	0.079
Total score	8.02 [3.87–12.16]	** < 0.001**

Data are derived from multilevel mixed-effects linear regression. Results are reported as estimated mean change and 95% confidence interval at 6-month visit in the T group. Bold data are intended to highlight statistically significant changes

*T* testosterone, *FSFI* Female Sexual Function Index, *CI* confidence interval

**Table 3:** Change over 6-months follow-up in the scores of FSFI domains in the T + E treatment group

FSFI domain	Estimated mean change [95% CI]	*p*
Desire	0.77 [0.32–1.22]	**0.001**
Pain	0.34 [− 0.01–0.70]	0.058
Arousal	0.50 [0.19–0.82]	**0.002**
Lubrication	0.64 [0.14–1.15]	**0.012**
Orgasm	0.63 [− 0.25–1.51]	0.160
Satisfaction	0.51 [− 0.01–1.04]	0.055
Total score	3.11 [1.31–4.92]	**0.001**

Data are derived from multilevel mixed-effects linear regression. Results are reported as estimated mean change and 95% confidence interval at 6-month visit in the T + E group. Bold data are intended to highlight statistically significant changes

*T* + *E* transdermal testosterone and local estrogens, *FSFI* Female Sexual Function Index, *CI* confidence interval

### Effect of testosterone treatment on cardio-metabolic parameters and clitoral area

We next evaluated the effect of transdermal T treatment on cardio-metabolic parameters to investigate its safety profile. No significant differences in the variations of total cholesterol and HDL, triglycerides, fasting glycemia, fasting insulin, and HbA1c levels were found after 6-month treatment in the group treated with T (*p* = 0.426, *p* = 0.069, *p* = 0.644, *p* = 0.078, *p* = 0.353 and *p* = 0.862, respectively) (Table 4).

**Table 4:** Change over 6-months follow-up in the cardio-metabolic parameters in the T treatment group

Cardio-metabolic parameters	Estimated mean change [95% CI]	p
Total cholesterol (mg/dL)	3.85 [− 5.62–13.31]	0.426
Triglycerides (mg/dL)	− 3.11 [− 16.31–10.09]	0.644
HDL-cholesterol (mg/dL)	− 4.17 [− 8.66–0.33]	0.069
Fasting glycemia (g/L)	− 0.12 [− 0.25–0.01]	0.078
HbA1c (mmol/mol)	0.05 [− 0.52–0.62]	0.862
Fasting insulin (mU/L)	− 0.59 [− 1.84–0.66]	0.353

Data are derived from multilevel mixed-effects linear regression. Results are reported as estimated mean change and 95% confidence interval at 6-month visit in the T group

*T* testosterone, *HDL* high-density lipoprotein, *HbA1c* glycated hemoglobin, *CI* confidence interval

Noteworthy, T treatment induced a significant increase in TT levels at 6-month visit vs. baseline (baseline TT: 0.70 nmol/L [0.50–1.21] vs. 6-month visit TT: 1.30 nmol/L [0.85–2.30], *p* = 0.04). However, we found an increase of clitoral area, as assessed using the planimetric method, which was not statically significant (0.46 cm^2^ [− 0.50; 1.43], *p* = 0.27). No hirsutism and acne or other adverse events were reported. In addition, SHBG did not show any significant change after treatment (*p* = 0.273).

## Discussion

The main finding of this pilot, retrospective study is that 6-month systemic treatment with transdermal T, alone or in combination with local estrogens, in women complaining for sexual symptoms, was associated with a significant change in the blood flow of clitoral corpora cavernosa arteries (assessed by CDU) and, specifically, with an increase in PSV. Noteworthy, in our study, clitoral CDU was performed in a resting condition, without any sexual stimulation, before and after 6-month T treatment, therefore, eliminating, or at least limiting, the potential bias of T central effects. To our knowledge, this is the first report to show a positive effect of T treatment on the increase in the PSV of the clitoral cavernous artery in FSD women. In men, this haemodynamic parameter is commonly evaluated in clinical practice and it is known to be positively modulated by T therapy [16, 17]. The increase (approximately 50%) in clitoral PSV observed in women undergoing systemic treatment with T, either alone or combined with estrogens, was not present in either non-hormonal or in estrogens-only groups. Moreover, our data show that 6-month systemic T treatment did not exert neither major side effects, nor alterations of the glycolipid profile.

Clitoral vascularization is fundamental to allow the physiological female genital arousal [37]. More recently, one of its most important parameters, PSV, has been proposed as a marker of arterial functioning [38, 39]. Indeed, genital arousal consists of clitoral tumescence and vaginal vasocongestion and swelling, which result from relaxation of endothelial SMCs [37]. It is also well known that clitoris is an androgens-responsive organ, throughout the entire life spectrum [40], both in its external and inner part, or clitourethrovaginal (CUV) complex [41]. For instance, during embryogenesis, androgens are fundamental for the development and morpho-functional regulation of genital organs [42, 43]. Thereafter, an important role of T in the regulation of genital arousal during adulthood has been also suggested [44]. However, the molecular mechanisms underlying this action of T on genitals, and in particular on clitoral function, remain partially unrevealed.

Preclinical studies have suggested that NO is the main modulator of clitoral blood flow, acting as a vasodilator through its second messenger cGMP [45–47]. We demonstrated that in vivo T treatment in ovariectomized rats upregulated the expression of several genes related to NO-mediated pathway in clitoral tissue, while improving acetylcholine (Ach)-induced relaxation, as compared to untreated ovariectomized rats [20]. Interestingly, we observed the same effects by treating ovariectomized rats with T plus the aromatase inhibitor letrozole, thus blocking its conversion into estradiol. In contrast, no significant modulation of either genes of NO-relaxant machinery or Ach responsiveness were found by treating ovariectomized rats with estradiol alone [20]. Consistently with these preclinical data, we could infer that, in women, T treatment could directly act on clitoral function and haemodynamic by improving the relaxant machinery of clitoral SMCs. Indeed, the human anterior vaginal wall, where the inner clitoris is embedded in, expresses the whole NO-dependent biochemical machinery (from synthases–NOS—to phosphodiesterases–PDE5) needed for sexual excitation in genital tissues [48]. Interestingly, both NOS and PDE5 are under T control [49, 50].

In line with the previously mentioned preclinical data in ovariectomized rats [20], we found that women treated only with estrogens showed no significant variation in clitoral PSV as compared to those undergoing non-hormonal treatment (even though a tendency towards a reduction was observed). In fact, in our preclinical studies in ovariectomized rats, in opposition to the permissive effect of T on clitoral SMCs relaxation, treatment with estradiol upregulated the expression of calcium-sensitizing, contractile pathway, of Ras homolog gene family member A (RhoA)—Rho-associated protein kinase (ROCK) [20]. The RhoA/ROCK pathway is one of the major contractile mechanisms in the vascular beds [51–53], thus suggesting that, in the clitoris, estradiol might positively modulate vasoconstriction more than vasorelaxation. However, in women undergoing combination therapy (systemic T + estrogens), T was still able to exert its positive effect on clitoral PSV. Treatment with local estrogens certainly represents one of the cornerstones of therapy against vaginal atrophy and dyspareunia due to estrogen deficiency, as it typically occurs in menopause [54]. Nevertheless, our data suggest a limited effect on the clitoris [20]. In addition, the tendency towards reduction in PSV observed in the E group could also have been due to the positive effect of estradiol on SHBG. However, intravaginal administration of estrogen was not able to increase SHBG levels in the present study, thus indicating a specific local effect.

Furthermore, no significant differences were found in the percentage of change of clitoral ACC and PI among the four treatment groups. This is probably due to the fact that clitoral PI was considered to specifically mirror atherosclerotic alterations in dysmetabolic conditions [21]. Clitoral Doppler parameters could be influenced by metabolic alterations, as previously published by our group [22]. In particular, we observed that metabolic syndrome, obesity and insulin resistance negatively affect those Doppler parameters [22]. However, in the present study insulin resistance and glyco-metabolic parameters, as well as BMI, were not different among the four subgroups. Nevertheless, the effect of T treatment on clitoral PI in a subset of FSD patients with metabolic disorders could merit further investigations.

In agreement with a positive effect of T on clitoral haemodynamics, a 6-month treatment with T alone or in combination with estrogens was associated with higher lubrication and arousal scores at FSFI questionnaire. All the vascular machinery underlying sexual arousal [37] and lubrication is, in fact, closely related to adequate genital vascularization [47]. A significant increase of FSFI desire, orgasm and total scores by T treatment was also observed, as compared to baseline. In this respect we recently demonstrated in animals, for the first time, that T effect on sexual appetite is mediated by a direct activation of AR, thus without any mandatory conversion into estradiol, as previously hypothesized [55]. In fact, in an experimental model of ovariectomized rats, treatment with DHT, a non aromatizable AR super-agonist, was able to induce appetitive and receptive behaviors [19].

Both groups treated with T also showed an increase in the FSFI satisfaction score, even though without reaching statistically significance. Sexual satisfaction is indeed a highly subjective parameter, more influenced by relational and psycho-social factors than from organic ones [56].

Systemic treatment with T also showed an improvement in the FSFI pain score, which was statistically significant in the T group, while only approaching statistical significance in the T + E group. This might be ascribable to the general improvement in all the other sexual domains, including, for example, lubrication. However, T might also exert its positive effects on pain sensitivity through a direct modulation of nociceptive mechanisms, as already demonstrated in several preclinical studies [57–60].

With regard to cardio-metabolic safety, no significant differences in the variations of total and HDL, triglycerides, fasting glycemia, fasting insulin, and HbA1c serum levels were found after T therapy. These results are consistent with those of a recent meta-analysis of randomized clinical trials (RCTs) aimed at assessing potential benefits and risks of T treatment for women [61]. When compared to placebo or to a comparator, T induced a significant rise in the amount of LDL and reduction in total cholesterol, HDL, and triglycerides, only when administered orally, but not transdermally [62]. Therefore, we should recognize the possible negative effects of T therapy in each patient, according to her metabolic and cardiovascular picture. In our study, T has been administered only for 6 months, but it is important to consider that therapies often could be prolonged, so that a careful evaluation is essential, as it happens for the administration of estrogens or estroprogestinic compounds. However, the relationship between endogenous T and cardiovascular (CV) health in women is still under debate [39]. In clinical research, higher androgen levels have been linked to an increased CV risk in women with polycystic ovary syndrome [62]. On the other hand, observational studies carried out on postmenopausal women demonstrated that physiological androgen levels are not associated with CV events or mortality [63, 64].

As suggested by the Endocrine Society Guideline, in our study we carefully prevented overdosing by measuring serum T levels upon a 6-month treatment [5]. We detected a significantly higher serum TT level when compared to baseline, paralleled with an increase (although not statistically significant) in clitoral area. No serious adverse events including newly diagnosed breast cancer, episodes of abnormal uterine bleeding and clitoromegaly were reported in the study. The absence of clitoromegaly is particularly relevant, since it can be considered as a marker of systemic T overtreatment. Noteworthy, both in postmenopausal women and in those in their fertile age, T excess also could produce other alterations to be considered as negative effects, such as acne, hirsutism, lowering of voice tone, and hair loss. Moreover, it should be recognized that in women with polycystic ovary syndrome who are hyperandrogenic, a reduced libido due to a reduced perception of their body, presenting with signs of hyperandrogenism, could be expected [65]. It should also be considered that breast neoplasms have often androgen receptors, but there are not available studies clarifying the possible role of androgens in this kind of tumors. Also to be mentioned is that liver function should be monitored throughout treatment.

Our study presents several limitations. The most obvious is its retrospective and observational nature, and the relatively small sample size. As a matter of fact, patients were not randomized to different treatment categories, but therapies were assigned based on their clinical presentation, thus generating a considerable bias. Second, TT was determined by electro-luminescence immunoassay and not by high-performance liquid chromatography-mass spectrometry, which is characterized by a better accuracy [66]. However, all the measurements were performed in the same central laboratory of a third level hospital, where the two analytic methods demonstrated a good agreement [67]. Third, our study population presented heterogeneity in its reproductive state. We tried to limit this bias by adjusting our analyses for known potential confounders, such as age and years since menopause. Finally, for the assessment of changes in orgasmic performance we did not use the unique psychometric tool validated to measure differences in intensity of female orgasm [68]. However, we partially supplied using the orgasmic domain of the FSFI. Another limitation is that we should have also studied a group of women treated with tibolone, characterized by both a certain androgenic and estrogenic activity. Such a comparison merits future investigation.

Nevertheless, this study presents some important strengths. First, we confirmed the role of the CDU, along with the static or dynamic morphological studies of the CUV complex [69, 70], in the evaluation of the clitoral vascularization and the sexual performance of this pivotal, but understudied organ. Second, we evaluated for the first time the effect of T treatment on haemodynamic ultrasound parameters, which were assessed in basal conditions. Therefore, our data help fulfilling a gender gap, by demonstrating a direct action of T on clitoris. Indeed, it has been reported that TRT in hypogonadal men with erectile dysfunction may restore penile haemodynamics by increasing cavernous artery PSV [16, 17, 71]. Third, patients included in the study underwent an adequate therapy follow-up (6 months), according to the 2014 Endocrine Society Clinical Practice Guideline [5] showing a good long-term efficacy and safety.

## Conclusions

In conclusion, the present study showed for the first time that a 6-month systemic T treatment, either alone or combined with estrogens, positively and directly modulated clitoral blood flow, also independently of sexual stimulation, in a small population of women complaining for sexual dysfunction. Moreover, systemic therapy with T was associated with a clinical general improvement in sexual function, as assessed by FSFI. Interestingly, no metabolic alterations or severe adverse events were detected in our study population at 6-month visit, suggesting that T treatment is characterized by a solid safety level. However, the topic of T treatment in women needs other more in-depth research and specifically designed randomized trials are needed to confirm our original findings.
